# Increased inflammation and brain glutamate define a subtype of depression with decreased regional homogeneity, impaired network integrity, and anhedonia

**DOI:** 10.1038/s41398-018-0241-4

**Published:** 2018-09-10

**Authors:** Ebrahim Haroon, Xiangchuan Chen, Zhihao Li, Thrusharth Patel, Bobbi J. Woolwine, Xiaoping P. Hu, Jennifer C. Felger, Andrew H. Miller

**Affiliations:** 1 Emory Behavioral Immunology Program, Winship Cancer Center, Emory University, Atlanta, GA 30322 USA; 2 Department of Psychiatry & Behavioral Sciences, Emory University School of Medicine, Atlanta, GA 30322 USA; 3 Wallace H. Coulter Department of Biomedical Engineering, Emory University & Georgia Institute of Technology, Atlanta, GA 30322 USA; 4 School of Psychology and Sociology and Shenzhen Key Laboratory of Affective and Social Cognitive Science, Shenzhen University, Shenzhen, 518060 Guangdong People’s Republic of China; 5 Shenzhen Key Laboratory of Affective and Social Cognitive Science, Shenzhen University, Shenzhen, 518060 Guangdong People’s Republic of China; 6 Department of Anesthesiology, Emory University School of Medicine, Atlanta, GA 30322 USA; 7 Department of Bioengineering, University of California, Riverside, CA 92521 USA

## Abstract

Combined increases in peripheral inflammation and brain glutamate may identify a subtype of depression with distinct neuroimaging signatures. Two contrasting subgroups of depressed subjects—with and without combined elevations in plasma C-reactive protein (CRP) and basal ganglia glutamate (high and low CRP-Glu, respectively) were identified by hierarchical clustering using plasma CRP (indexing peripheral inflammation) and magnetic resonance spectroscopy (MRS)-based measurement of left basal ganglia glutamate. High CRP-Glu group status was associated with greater severity of anhedonia and cognitive and motor slowing. Local- and network-level measures of functional integrity were determined using brain oxygen level-dependent (BOLD)-oscillatory activity and graph theory. Greater decreases in concordance of oscillatory activity between neighboring voxels (Regional Homogeneity ‘ReHo’, *p* < 0.01) within the MRS volume-of-interest was associated with the High CRP-Glu subgroup. Using brain-wide, CRP-Glu ReHo contrast maps, a covariance network of 41 regions-of-interest (ROIs) with similar ReHo decreases was identified in the High CRP-Glu group and was located to brain structures previously implicated in depression. The 41-ROI network was further decomposed into four subnetworks. ReHo decreases within Subnetwork4—comprised of reward processing regions —was associated with anhedonia. Subnetwork4 ReHo also predicted decreased network integrity, which mediated the link between local ReHo and anhedonia in the Low but not High CRP-Glu group. These findings suggest that decreased ReHo and related disruptions in network integrity may reflect toxic effects of inflammation-induced increases in extrasynaptic glutamate signaling. Moreover, local BOLD oscillatory activity as reflected in ReHo might be a useful measure of target-engagement in the brain for treatment of inflammation-induced behaviors.

## Introduction

Research on major depressive disorder (MDD) has been stymied by within- and between-subject variability. One approach to limiting this variability has been to identify patient subgroups based on pathophysiological processes such as increased inflammation^1–3^. Indeed, a distinct subtype of depression characterized by increased inflammation has been proposed^4^. In the brain, inflammation triggers a broad array of neurotransmitter, molecular, neural, and behavioral effects^5–10^. For example, altered glutamate (Glu) neurotransmission in subcortical regions has been a key neurochemical target of inflammation in depression^11^. Previously published data indicate that increased plasma concentrations of the systemic inflammatory marker c-reactive protein (CRP) among subjects with MDD predicted elevated Glu concentrations in left basal ganglia regions as measured by magnetic resonance spectroscopy (MRS), which in turn predicted greater anhedonia and decreased psychomotor speed, reaction-time, and information processing^12^. Similar results were found among individuals treated with the inflammatory cytokine interferon-alpha^12–14^.

As to the mechanisms by which inflammation influences Glu neurotransmission, inflammatory cytokines increase spillover of Glu from the intra- into the extrasynaptic space by decreasing the ability of astrocytes to clear, buffer and contain Glu, while concurrently impairing mechanisms that remove Glu from the extracellular matrix^15,16^. In addition, glial cells and trafficking macrophages increase surface expression of cystine/glutamate exchanger (Xc)-transporters that extrude Glu into the extrasynaptic space in exchange for cystine^11,16,17^. Of note, these Xc- transporters release large volumes of Glu in dangerously close proximity to extrasynaptic receptor-binding sites^18^. Finally, immune activation increases Glu-like molecules such as quinolinic acid, which can promote glial Glu release or impair its reuptake^19,20^. Taken together, inflammation preferentially increases Glu in extrasynaptic compared to intrasynaptic locations^15,18,21–23^. Extrasynaptic Glu freely diffuses into the extracellular space and binds to extrasynaptic *N*-methyl-d-aspartate (NMDA) receptors, leading to chaotic, noisy, incoherent signaling activity in the short-term, and synaptic toxicity by suppressing intracellular survival mechanisms in the long-term^18,24–26^. Nonetheless, neural activity-based biosignatures associated with inflammation-induced Glu dysregulation are not well-characterized.

A primary objective of this work was to identify and contrast two subgroups of depressed subjects with and without combined elevations in plasma CRP and basal ganglia glutamate and examine their impact on neural activity. Investigating neural activity using oscillatory frequency, amplitude or coherence of spontaneous brain oxygen level-dependent (BOLD)-fluctuations during resting-state functional MRI offers a unique opportunity to examine these associations^27–29^. Regional Homogeneity (ReHo)—an activity-based metric of concordance in BOLD-signal fluctuations between neighboring voxels—is an effective estimate of local activity coherence at millimetric resolution^29–32^. Based on the hypothesis that combined increases in inflammation and extrasynaptic Glu can lead to disorderly, incoherent local neural activity, we predicted that concurrent increases in peripheral inflammation and MRS Glu would identify a subgroup of depressed patients with reduced local ReHo in the basal ganglia and potentially other brain regions. Moreover, we hypothesized that decreased ReHo in regions affiliated with reward, salience, and attention would be associated with anhedonia and psychomotor slowing. Finally, using exploratory path analyses, we examined whether altered network-level interactions among the above regions mediate the link between inflammation, Glu, local ReHo, and anhedonia.

## Materials and methods

### Subjects

Fifty subjects with a diagnosis of MDD confirmed using Structured Clinical Interview for DSM-IV (SCID) and with ages ranging between 21–65 years were screened for inclusion into the study^33,34^. Bipolar depressed subjects were also included due to shared mechanisms of anhedonia, inflammation and Glu dysregulation^35^. Exclusion criteria included unstable medical conditions; cognitive, psychotic or substance abuse disorders; and intake of any psychotropic or immune-altering medications. Detailed information on subject recruitment and evaluation is provided in Supplementary Methods. All participants signed informed consent, and the study was approved *a priori* by the Institutional Review Board of Emory University. Subjects presented in this manuscript represent a subset of those recruited for NCT01426997.

### Behavioral and cognitive assessments

Depression severity was measured using the Inventory of Depressive Symptoms-Self Reported (IDS-SR)^36,37^. Anhedonia was measured using a 3-item subscale derived from IDS-SR items #8, 19, and 21 (‘‘anhedonia’’) that has been previously validated in other studies^12,38,39^. Psychomotor and cognitive performance were assessed using the Finger Tapping Test^40^; Trails Making Test A (TMT)^41^; Digit Symbol Substitution Test of Wechsler Adult Intelligence Scale^42^; Movement and Reaction times—Simple (SMT, SRT, respectively) and Five-Choice Reaction Time (5CMT, 5CRT, respectively)—and “Stockings of Cambridge” (SOC) Tasks of the Cambridge Neuropsychological Test Automated Battery (CANTAB)^43,44^. Of note, principal components analysis of the SOC yielded performance scores for three dimensions (SOC1, SOC2, SOC3) corresponding to ‘‘mean initial thinking time’’, ‘‘mean moves’’, and ‘‘mean subsequent thinking time’’, respectively (Supplementary Methods).

### C-reactive protein

Blood was sampled between 8 and 10:00 a.m. to limit circadian variation, and plasma was collected and stored at −80 °C for batched assay^12,39^. Hs-CRP was measured by the immunoturbidometric method using the Beckman AU 480 chemistry analyzer and Ultra WR CRP reagent kit (Sekisui Diagnostics, LLC, Lexington, MA, USA). Mean inter- and intra-assay coefficients of variation were reliably < 10%. CRP values were log-transformed for normality.

### MRI methods

#### **MRI methods**

All scans were acquired using Siemens 3 T Trim-Trio systems (Siemens Medical Systems, Erlangen, Germany).

#### **Acquisition**

Anatomical T1 images were obtained using three-dimensional magnetization- prepared rapid gradient-echo with settings of repetition time (TR) = 2300 milliseconds (ms), echo time (TE) = 3.02 ms, time following inversion (TI) = 1100 ms, flip angle = 8^o^ and voxel size 1 × 1 × 1 mm^3^. Single-voxel MRS data were acquired using a PRESS (point-resolved spectroscopy) technique with TR = 3000 ms, TE = 30 ms, sampling size = 1024, 128 averages. A rectangular voxel sized 20 × 40 × 30 mm^3^ located on the left basal ganglia was used to obtain single-voxel ^1^H-MRS^12,13^. Details of acquisition and voxel location are provided in Supplementary Methods.

#### Resting-state fMRI images

Resting-state fMRI images were acquired using a Z-saga EPI-pulse sequence for recovering ventral-frontal signal losses regularly seen in gradient-echo BOLD fMRI at 3.4 × 3.4 × 4 mm^3^ resolution in 30 × 4-mm-thick axial slices with the following parameters: FOV = 220 mm, TR = 2950 ms, TE1/TE2 = 30/67 ms, FA = 90^o^, scan time = 7.4 min (150 repetitions). Forty-five Subjects were required to look at a fixation cross during scanning.

#### Post processing MRS data-estimation of absolute Glu concentrations

MRS metabolites were estimated with water-scaling in LC Model using following settings: spectral bandwidth 0.2–4.0 ppm and 2048 complex points^46^ and corrected for cerebrospinal fluid (CSF) volume fraction using methods published earlier^12^. Methods used for post processing including voxel tissue composition, spectral quality, and absolute quantitation of metabolites are provided in Supplementary Table 1 and Supplementary Methods.

### Analysis of resting-state data

#### **Preprocessing procedure**

Signal spike, slice-timing shift, and motion were corrected in AFNI (https://afni.nimh.nih.gov)^47^, followed by removal of motion and CSF signals within a general linear model (GLM). Local BOLD oscillatory measures were calculated with the residual time-series data derived from the GLM. Of note, white matter (WM) signal was not removed because WM is a key target of inflammation, Glu dysregulation, and depression^48,49^.

#### Group comparisons of local BOLD oscillatory data

Local BOLD oscillatory measures—including ReHo, amplitude of low-frequency fluctuations (ALFF) and resting-state fluctuation amplitude (RSFA)—and their fractionated derivatives were obtained and compared. ReHo is a metric that estimates the concordance (statistical similarity of spontaneous neural activity) between a given voxel and its spatially adjacent, neighboring voxels within the context of a given time-series^29,32^. ALFF is an amplitude-based index of local oscillations in low-frequency range ( < 0.01–0.1 hz)^50,51^ and RSFA utilizes power spectral analysis to estimate vascular reactivity information in spontaneous BOLD activity^28^. Oscillatory data were analyzed in two ways: (1) Volume-of-interest (VOI)-based analysis: Averages were calculated across all voxels in the left BG MRS VOI for individual subjects and compared between the groups and (2) Brain-wide analysis of BOLD oscillatory activity that differed in the VOI-based analysis. For each subject, the ReHo data were converted into standard MNI space and spatially smoothed with FWHM = 4 mm. Group comparisons were performed using AFNI “3dttest++”, generating a ReHo-difference map after correcting for multiple-test false-positive errors using Monte Carlo serial simulation followed by *p*-value thresholds specified in recent publications addressing the issue of multiple comparisons (“Clustsim” option, *p*_voxel_ ≤ 0.01, *p*_cluster_ ≤ 0.05) ^52,53^.

#### Network analysis

For each subject, the residual time-series data were temporally filtered (0.01–0.1 Hz), converted into MNI space and spatially smoothed (FWHM = 4 mm). Average time-series data were calculated for regions-of-interest (ROIs). Cross-correlation analysis among time-series data was preformed using MATLAB (MathWorks, Natick, MA, USA), generating a correlation-coefficient matrix which was further transformed into a Fisher *z*-score matrix. With the *z*-score matrix, graph-theory-based measures of brain connectivity were estimated using the Brain Connectivity Toolbox in MATLAB (https://sites.google.com/site/bctnet)^54,55^. Networks were defined as a collection of nodes (ReHo ROIs), and links (edges) between pairs of ROIs. Weighted, undirected network measures were obtained using adjacency and distance matrices within the brain connectivity toolbox. Eleven measures of network integrity including degree (positive and negative connection strengths); internodal clustering (positive and negative clustering coefficients, transitivity, and modularity); assortativity; distance (path length, global efficiency) and eccentricity (diameter and radius) were measured (details in Supplementary Methods). Using graph theory-based metrics enabled computation of integrity at network and subnetwork-level. In addition, ‘‘average connectivity *z*-scores’’ were also calculated across groups of ROIs analysis by averaging the time-series.

### Statistical methods

**Fig. 1:**
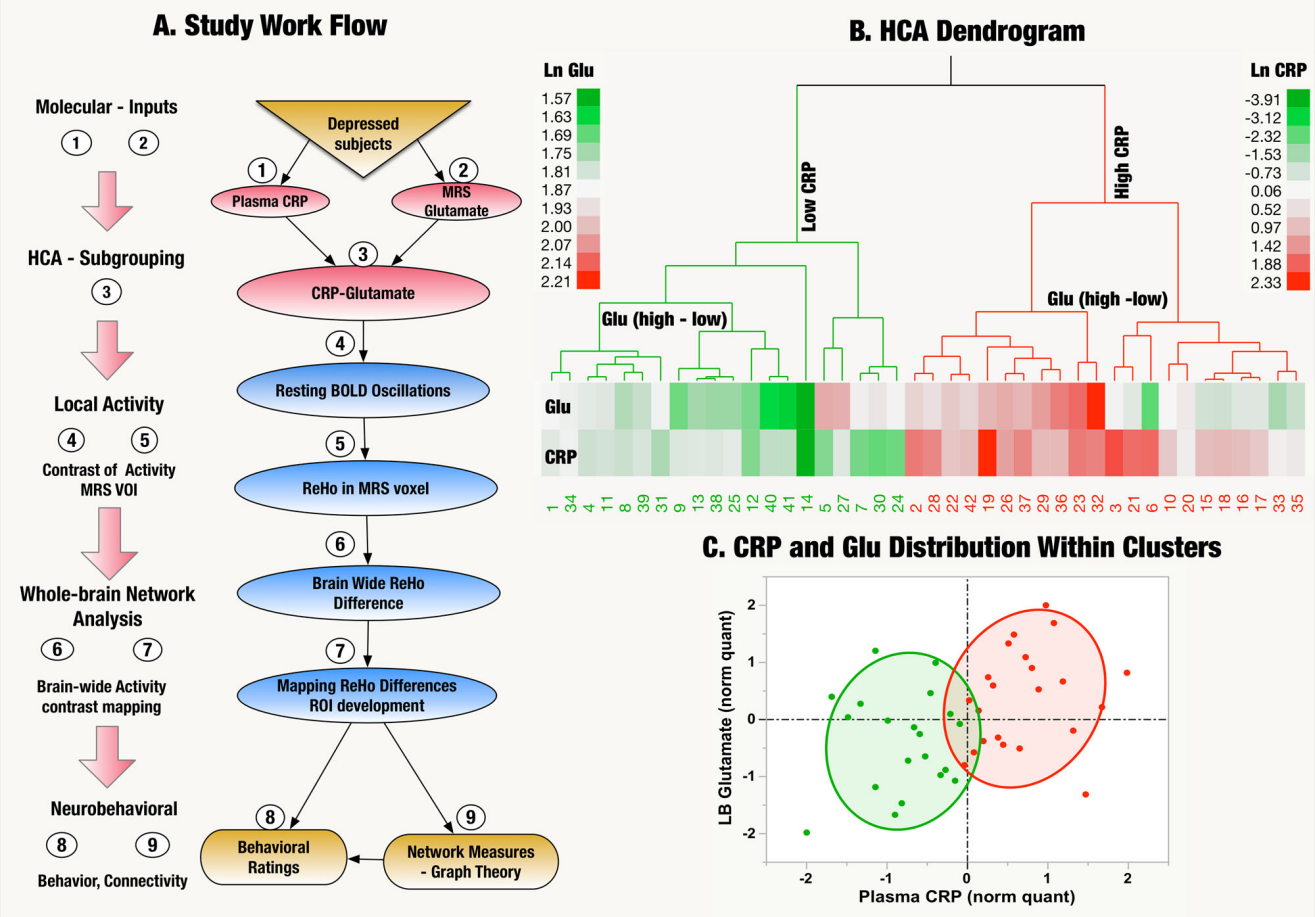
Overview of analytic pipeline and group assignments. **a** Study Work Flow: Data processing and analytic workflow: BOLD brain oxygen level dependent, MRS magnetic resonance spectroscopy, HCA hierarchical cluster analysis, VOI volume-of-interest, CRP plasma C-reactive protein, ReHo regional homogeneity, ROI region-of-interest. **b** Dendrogram/heatmap of hierarchical clustering analysis (HCA): Using HCA, the sample was divided into High (*n* = 22) and Low (*n* = 19) plasma C-reactive protein (CRP)-glutamate (Glu) groups. The accompanying tree-shaped dendrogram and Heatmap illustrate the clustering algorithm. In HCA, the data is arranged hierarchically with log-normalized (Ln) CRP as first node of the hierarchy followed by Ln Left Basal (Glu) as the secondary node. The agglomerative nature of the method used where minor clusters are progressively combined to yield larger clusters is also depicted as branches of the dendrogram. The length of dendrogram lines are proportional to cluster distances. The Legends along the side of the dendrogram provide the range of individual subject measures of the variables used (Ln CRP and Ln LB Glu) color-coded as cool (green)-warm (red) colors. **c** CRP and Glu distribution: The scatterplot demonstrates the distribution of the association between CRP and LB Glu in the High vs. Low CRP-Glu groups. Shaded 80% confidence-interval ellipses are used to represent High (red) and Low (green) CRP-Glu groups (respectively). Values of CRP and LB Glu in *x* and *y*-axis (respectively), were quantile-normalized (norm quant) for easy visual inspection and color-coded for the two groups of interest. The broken lines represent the median values. Using median-split 2/3rd of subjects were classified as having both high CRP/high glutamate in contrast to 1/3rd of subjects with high glutamate who did not fall into the high CRP group (Chi Sq = 4.7, *p* = 0.03). Thus, the association between CRP and glutamate was neither absolute or invariable with neither qualifying to be a proxy for the other

Agglomerative Hierarchical Cluster Analysis (HCA) using Ward’s linkage method in JMP Pro v13 for Mac (SAS Institute, Cary, NC, USA) was used to classify subjects based on plasma CRP and MRS-based measures of basal ganglia Glu. Both values were log-transformed to scale for uniformity and normality. Cubic Clustering Criterion (CCC—a measure of the cluster numbers providing the best fit) and evaluation of ‘screen’ plots were used to inform the number of groups. Both CCC (–1.891 for 2 cluster solutions vs. −2.88, −2.61, −2.13 for 3, 4, and 5 cluster solutions, respectively) and screen plot revealed a two-cluster solution as the most parsimonious model. A dendrogram depicting the cluster differentiation is provided in Fig. 1b. ‘‘Variable clustering” in JMP Pro (similar to ‘‘varclus’’ in SAS) was also used to identify groupings of variables based on shared brain signatures^56^. Data were checked for univariate and multivariate normality using standard procedures. Comparisons of background variables were performed using *t*-tests and chi square, and variables that were different between High and Low CRP-Glu groups were controlled as covariates in further analysis. Canonical, stepwise, discriminant function analysis (DFA) was used to predict membership in High vs. Low CRP-Glu groups using a categorical classifier (*x* variable = CRP-Glu status) on known continuous variables (‘‘*y*’’ responses). Adaptive elastic regression models in JMP Pro were used to test predictive associations owing to their ability to shrink covariances, limit collinearity, and decrease overall prediction error. Following variable selection (within elastic net models), the predictors and covariates were imported into standard least squares regression models to test for multiple comparisons (using False Discovery Rate corrected (FDR)-*p*-values) and to measure statistical power and estimate effect sizes. Effect sizes were measured using Cohen’s *d* (95% CI)^57^, and alternate measures (*F*-statistic, partial eta, omega, r-squared) were converted into ‘‘d’’ for consistency. Path analysis was performed using Structural Equation Modeling (SEM) protocol in STATA (College Park, Texas, USA).

## Results

### Sample characteristics

Of the 50 subjects recruited, eight were excluded [six with poor quality MRS data (metabolite variances > 20% or motion artifacts, one due to excessive head motion and one for whom resting-state fMRI was not available]. Data from the remaining 42 patients were included in the analyses. Figure 1a illustrates the analytic pipeline used for the study. Using HCA, two clusters—High (*n* = 22) vs. Low (*n* = 20) CRP-Glu groups were identified (Fig. 1b). Both plasma CRP and absolute left basal ganglia Glu were significantly elevated in the High vs. Low CRP-Glu group (*p* < 0.001 and 0.002, respectively). Of note, the cross-tabulation of subjects classified into ‘‘high’’ and ‘‘low’’ groups using + above/-below median of CRP and glutamate indicated that 66% of individuals with high glutamate fell into the high CRP group, while the remaining 33% of subjects with high glutamate were classified into the low CRP group (Fig. 1c) (Likelihood ratio = 4.76, *p* = 0.03). Thus, the association between CRP and glutamate was neither absolute or invariable with neither qualifying to be a proxy for the other. The samples were well-matched on all clinical, demographic variables and (right vs. left) handedness other than BMI, which was significantly higher among High vs. Low CRP-Glu subjects (*p* < 0.001). BMI was entered as a covariate in all subsequent analyses (unless otherwise specified). Only 3/42 subjects were SCID + bipolar depression with 1 and 2 in Low and High CRP-Glu groups, respectively, (Table 1 and Supplementary Table 2).

**Table 1:** Demographic and clinical characteristics of the CRP-Glu groups

Group statisticsMean ± SD	Low CRP-GLU Cluster (*n* = 20)	High CRP-GLU Cluster (*n* = 22)	Test, *p*
Background and clinical			
Age	38.5 ± 11.0 (122.2)	37.9 ± 11.3 (127.9)	*F*(1,40) = 0.3, *p* = 0.86
Sex: (Females) %	12 (40%)	18 (60%)	Chi Sq = 2.44, *p* = 0.12
Race: African Americans, (%)	11 (44%)	14 (56%)	Chi Sq = 0.32, *p* = 0.57
Education: College grads (%)	9 (45%)	8 (36%)	Chi Sq = 0.32, *p* = 0.57
Smoker (%)	6 (30%)	4 (19%)	Chi Sq = 0.81, *p* = 0.37
BMI: Mean (SD)	26.63 ± 4.29	35.66 ± 7.87	*F*(1,40) = 20.7, *p* < 0.001^a^
Bipolar/unipolar depression	1/19	1/21	Chi Sq = 0.005, *p*=0.94
Duration of current episode of depression (months)	170.90 ± 172.99	204.41 ± 153.27 (23491.87)	*F*(1,40) = 0.44, *p* = 0.51
Age of onset of depression (Years)	21.80 ± 12.67	16.95 ± 16.95	*F*(1,40) = 1.99, *p* = 0.17
Number of depressive episodes	1.89 ± 3.02	1.38 ± 0.97	*F*(1,38) = 0.55, *p* = 0.46
Number of antidepressants used in current episode	0.95 ± 2.01	0.73 ± 1.16	*F*(1,40) = 0.20, *p* = 0.66
Behavioral			
Anhedonia (IDS3-items)	4.20 ± 1.93	5.50 ± 1.71	*F*(1,40) = 5.3, *p* = 0.03^a^
Response to good/desired events (IDS-SR Item #8)	1.25 ± 0.85	1.82 ± 0.66	*F*(1,40) = 5.88, *p* = 0.02^a^
Leaden paralysis/physical energy (IDS-SR Item #30)	0.95 ± 0.69	1.64 ± 0.95	*F*(1,40) = 7.04, *p* = 0.01^a^
IDS-SR-Total	34.35 ± 7.5	38.2 ± 8.0	*F*(1,40) = 2.6, *p* = 0.12
Cognitive			
Trails (total score)	28.64 ± 9.73	38.69 ± 17.38	*F*(1,40) = 5.20, *p* = 0.03^a^
Sock of Cambridge (initial think time—principal component)	-0.62 ± 0.81	0.56 ± 1.80	*F*(1,40) = 7.19, *p* = 0.01^a^
CANTAB 5-choice movement Time (msec)	468.67 ± 83.49	554.27 ± 117.29	*F*(1,40) = 7.29, *p* = 0.01^a^
Neurochemical			
Left basal ganglia glutamate	6.05 ± 0.68	6.93 ± 0.96	*F*(1,40) = 11.2, *p* = 0.002^a^
Immune			
Plasma CRP (mg/L)	0.39 ± 0.23 (*n* = 17)	3.99 ± 2.27 (*n* = 20)	*F*(1,35) = 49.6, *p* < 0.001^a^

*BMI* body mass index, *IDS-SR* Inventory for Depressive Symptoms-Self-Rated Version, *anhedonia* 3-item Anhedonia Subscale, *CANTAB*– Cambridge, *CRP* c-reactive protein (plasma)

^a^ Significant ANOVA

### Behavioral and cognitive variables

DFA with post hoc analysis of variance (ANOVA) indicated that anhedonia [*F*(1,40) = 5.34, *p* = 0.03], 5CMT [*F*(1,40) = 7.29, *p* = 0.01], TMT [*F*(1,40) = 5.20, *p* = 0.03] and SOC1 [*F*(1,40) = 7.19, *p* = 0.01] were significantly associated with High CRP-Glu status **(**Table 1**)**. Analysis of individual IDS-SR items indicated that Item#8, signifying positive valence [“Response of your mood to good or desired events”: *F*(1,40) = 5.53, *p* = 0.02] and item#30 [Leaden Paralysis/Physical Energy: *F*(1,37) = 5.80, *p* = 0.02] were also associated with High CRP-Glu status. Most the effect sizes were in the ‘‘large’’ range (*d* > 0.8).

### Effect of CRP-Glu status on BOLD-signal metrics in MRS voxel

**Fig. 2:**
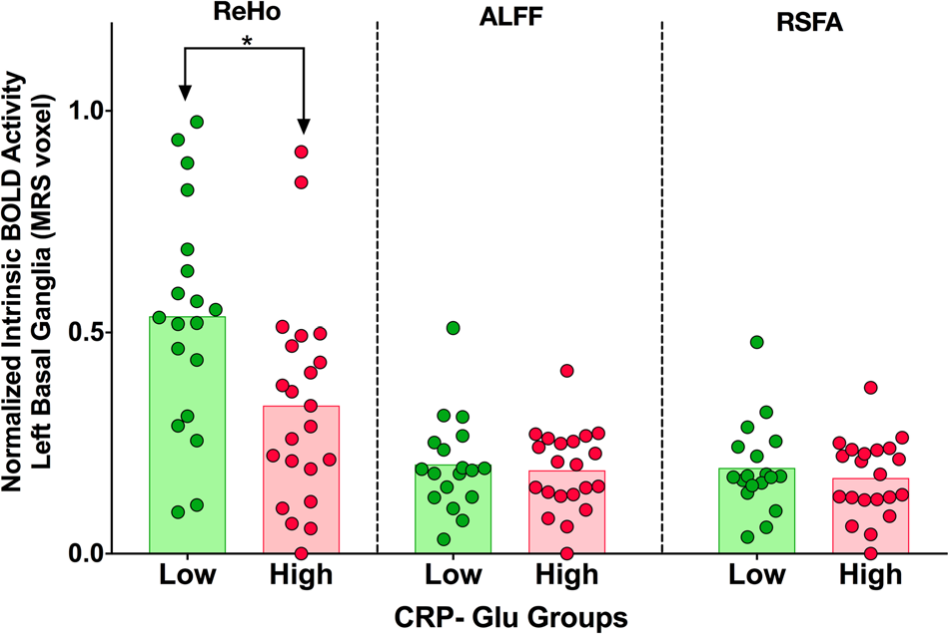
Local Activity Contrast in the MRS Voxel-of- Interest (VOI). Scatterplot with bar demonstrating differences in plasma CRP and glutamate in the left basal ganglia between High vs. Low CRP-Glu groups. The upper end of the bar is the mean and shaded regions represent range between mean and 0. Green shade represents Low CRP-Glu and red shades represent High CRP-Glu. Comparison of group means of the three bold oxygen level-dependent (BOLD) indices between (Low-High) CRP-Glu groups revealed that Regional Homogeneity (ReHo) was the only metric of BOLD oscillatory activity that significantly differed between groups: (ReHo). Table 2 provides a detailed summary of individual means and SD

DFA with post hoc ANOVA identified reductions in ReHo in the left basal ganglia MRS VOI as the local BOLD oscillatory measure associated with High CRP-Glu status [*F*(1,40) = 10.20, *p* = 0.003, canonical *r* = 0.45]; with leave-one-out cross-validation indicating a classification-accuracy of 76.2% (Fig. 2 and Supplementary Table 2). Owing to a lack of similar association, ALFF and RSFA were excluded from further analysis. Testing of 3-way interactions between CRP and glutamate along with covariates (age, sex, race, BMI, smoking) confirmed that neither CRP nor glutamate alone (both *p* > 0.05) but only (Low-High) CRP-Glu contrast [PE = 0.83 (0.34–1.33), Wald Chi Sq = 10.8, *p* = 0.001] predicted ReHo. Of note, the same elastic net model also revealed that race [African American-Caucasian American = –0.57(–1.08– –0.06), Wald = 4.82, *p* = 0.03] had a significant impact on ReHo decreases and hence was controlled as a covariate in all subsequent analyses.

### **Whole-brain ReHo a**nd ReHo subnetwork partitioning

**Fig. 3:**
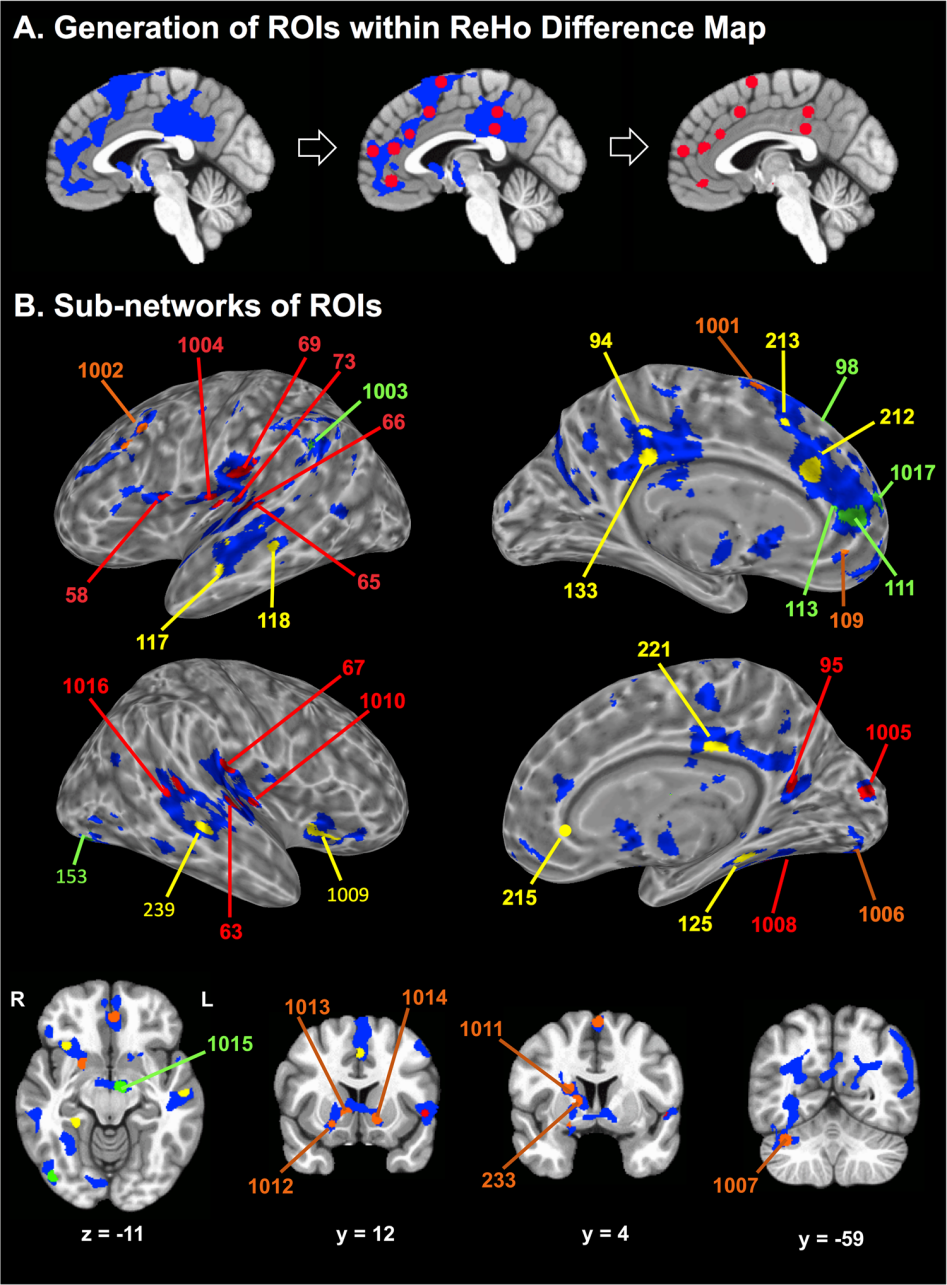
Regions-of-interest (ROIs) within the ReHo-difference map. **a** Generation of ROIs (right: red spheres) within the ReHo-difference map (left: blue patches): Overlapped portions between the ReHo-difference map and 10 mm diameter spheres (middle: red circles on blue regions). The background image is a sagittal slice of the MNI-152 brain atlas. **b** Four sub-networks (SBNs) of ROIs shown on the cortical surface (top, middle) and slices (bottom) of the MNI-152 brain. The ROI numbers and their corresponding brain structures are identified in Supplementary Table 3

To reliably locate whole-brain ReHo changes within functionally defined space, 10 mm spheres (#283) were placed on previously published functional coordinates^56,58,59^, and the spheres demonstrating > 50% overlap with ReHo-difference map were included as ROIs. A total of 41 ROIs—27 from the first set of 264 ROIs^58^ and an additional set of 17 ROIs curated from depression-related studies^56,59^ that fulfilled the “overlap’’ criteria were included for further analysis (Fig. 3). Variable clustering approaches were used to decompose the large number of ROIs (#41) into four subnetworks of ROIs. A detailed description of the ROIs including their coordinates and subnetwork affiliations are provided in Supplementary Table 3. ROI masks were applied to calculate ‘‘mean ReHo” of each subnetwork for each individual and grouped for further analyses.

### Subnetwork4 ReHo and behavioral associations

Subnetwork4 ReHo was the only predictor of anhedonia [PE = –20.8, *t* = –3.76, FDR *p* < 0.001, *d* = 1.22 (0.55–1.84)] and IDS-SR-positive valence Item#8 [PE = –18.1, *t* = –3.05, FDR *p* = 0.004, *d* = 1.03 (0.33–1.63]. Of note, Subnetwork4 included several ROIs within canonical reward and salience networks including ventromedial prefrontal (vMPFC), and dorsal (DS) and ventral striatal (VS) regions (Fig. 3 and Supplementary Table 3)^56,59,60^. None of the other three subnetworks demonstrated significant associations with anhedonia, although Subnetwork1 ReHo was predictor of psychomotor processing speed as measured by TMT [PE = –0.41, *t* = –2.71, *p* = 0.01, *d* = 0.85 (0.19–1.46) and Subnetwork2 ReHo was predictor of IDS-SR leaden paralysis Item#30 [PE = –0.35, *t* = –2.38, *p* < 0.02, *d* = 0.74 (0.19–1.34)].

### Network correlates of ReHo changes

**Fig. 4:**
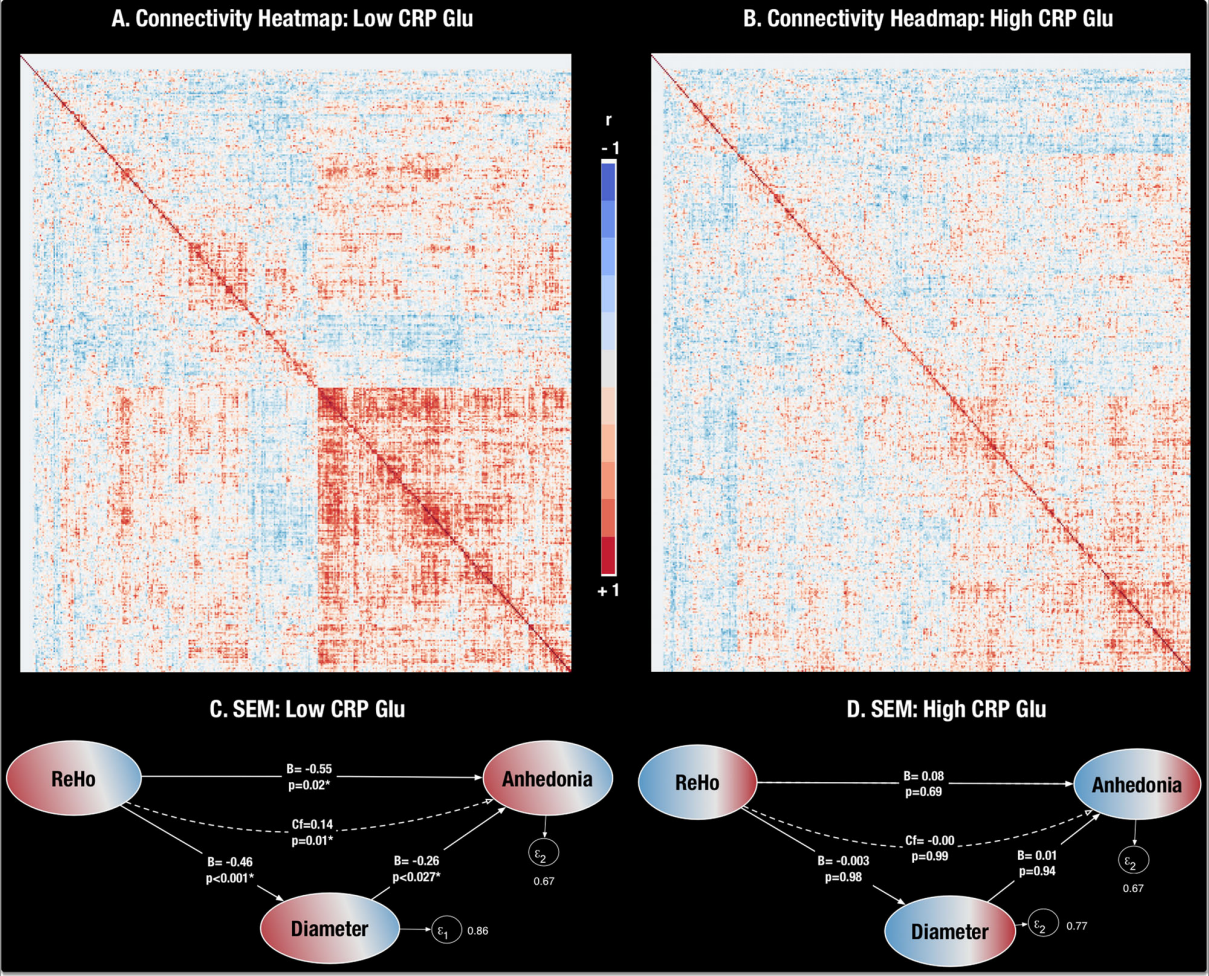
Decreased ReHo is associated with decreased network integrity **a**, **b**. Clustered correlation maps of grouped Fisher-z-connectivity scores: Heatmap demonstrating clustered cell plot of correlations in functional connectivity (Fisher *z*-scores) between all 41 ReHo-ROIs in the Low CRP-Glu (**a**) and in the High CRP-Glu grouping (**b**). Correlations in functional connectivity (Fisher *z*-transformed scores) between all 41 ReHo ROI-seeds are depicted as ranging from red ( + 1) to blue (–1). **c, d**. Path Analysis of ReHo, Network Measures and Anhedonia: Path Analysis of ReHo, Network Measures and Anhedonia: A significant effect for the path extending from Subnetwork4 ReHo → network eccentricity (diameter, radius) → anhedonia was seen only in the Low [diameter path coef (95% CI) = 0.14 (0.33–0.24), *z* = 2.58, *p* = 0.01] (**c**) but not in High CRP-Glu groups (both *p* > 0.05) (**d**)

Clustered cell plots of correlations in functional connectivity (Fisher *z*-scores) between all 41 ReHo-ROIs in High and Low CRP-Glu groups are demonstrated in Fig. 4a, b. Significant differences were noted in correlation- and covariance-based comparisons of Fisher-*z*-connectivity matrix between High and Low CRP-Glu groups [Jennrich’s Chi Sq(820) = 2235.65 and Box Chi Sq(861) = 2645.61, respectively, both *p* < 0.001]. A MANOVA model with post hoc Benjamini-Hochberg correction^61^ identified significant but modest reductions in “global efficiency” (reflecting greater network dysconnectivity) of a putative network combining all 41 ROIs measured in the High vs. Low CRP-Glu group [mean (95% CI) = 0.22 (0.20–0.24) vs. 0.25 (0.23–0.27), *t*(34) = -2.10, *p* < 0.04, *d* = 0.66 (0.03–1.280)].

### Disruptions in Subnetwork4 architecture associated with ReHo changes

Multivariate ANCOVA followed by post hoc Benjamini-Hochberg correction indicated that Subnetwork4 mean ReHo positively predicted global efficiency and node strength (*p*-corr = 0.009 and 0.02, respectively) and negatively predicted network diameter, path length and radius (*p*-corr = 0.01, 0.01, and 0.03, respectively) in the groups combined (Supplementary Table 4). Subnetwork4 assortativity (indicating decreasing network resilience) was also associated with psychomotor slowing indexed by prolongation of 5CMT [PE = 0.47, *t* = 3.46, FDR *p* = 0.004, *d* = 0.77 (0.09–1.40)]. Network eccentricity measures, i.e., diameter and radius (reflecting greater nodal segregation) positively predicted severity of scores on IDS-SR item#30 “paralysis” [PE = 1.01, *t* = 3.55, FDR *p* < 0.001, *d* = 1.18 (0.60–1.84) and PE -1.02, *t* = –3.6, FDR *p* < 0.001, *d* = 1.24 (0.55–1.88) for diameter and radius, respectively].

### Exploratory path analysis linking CRP-Glu, ReHo, network metrics, and behavior

A path analysis based on Structural Equation Modeling was used to examine links among ReHo, network metrics and anhedonia. The pathway from Subnetwork4 ReHo → Subnetwork4 measure → anhedonia was examined independently in the combined (both), and the High and Low CRP-Glu groups separately (Fig. 4c, d). A significant effect for the path extending from Subnetwork4 ReHo → network eccentricity (diameter, radius) → anhedonia was seen only in the Low [diameter path coef (95% CI) = 0.14 (0.33–0.24), *z* = 2.58, *p* = 0.01, and radius = [0.13 (0.006–0.26, *z* = 2.06, *p* = 0.04)] but not among High or combined CRP-Glu groups (both *p* > 0.05). SEM models replacing diameter with radius yielded identical results, while other measures associated with Subnetwork4 ReHo (global efficiency, network strength, path length) did not demonstrate statistically meaningful mediation effects.

## Discussion

A distinct subtype of depressive behaviors including anhedonia and psychomotor slowing was revealed by CRP-Glu status. Behavior is an emergent property of coherent network activity patterns, and symptoms represent a failure of this function. It is unclear, if the lower limit of a critical minimum threshold of ordered, coherent local activity necessary to maintain network homeostasis is breached in the High (but not Low) CRP-Glu group. As noted earlier, a disrupted balance between intra- vs. extrasynaptic signaling is likely to underlie the reduction in local homogeneity in the High CRP-Glu group ^18,24,62^.

Stressed astroglial cells functioning under the duress of immune stimulation might lie at the epicenter of this network imbalance. Astroglial cells exist in assemblies that act as hubs that connect closely located but functionally divergent circuits (e.g., reward vs. aversion) by cross-synaptic dissemination of tonic, coherent, “near threshold” stimulation via their gliotransmission capabilities^63^. The association between this tonic, coherent activity and local homogeneity is unclear but worth exploring. MRS primarily measures intracellular (astroglial) glutamate pool and hence increases in these signals might reflect increased cycling of glutamate or excessive intracellular pooling due to progressively declining astrocytic function^64,65^. Of note, our previous MRS data in depressed patients indicate increased basal ganglia myo-inositol (a putative marker of astroglial function) in association with increased CRP, possibly indicating a relationship between inflammation and astroglial dysfunction ^12^.

Strengths of this study include use of multi-modal imaging techniques to compare key local and long-distance metrics of functional brain activity in a well-characterized, unmedicated group of subjects. Nevertheless, several limitations of the study need to be considered. Based on the aims and hypotheses, attention was focused on MRS glutamate signals from the left basal ganglia region—excluding other ROIs and other metabolites. However, a group comparison of the various other MRS metabolites did not reveal any group differences (Supplementary Table 2). Use of methods that estimate glutamine, lactate and glutamine-glutamate cycling such as ^13^carbon, hyperpolarized or ultra-high field MRS might have enabled a better appreciation of the underlying bioenergetic changes and helped discriminate glutamate dysregulation induced by astrocytic vs. neuronal pathologies. In addition, MRS provides limited spatial resolution that does not enable precise information about synaptic location or activity. However, MRS glutamate signals have consistently predicted neural activity in task- and transcranial magnetic stimulation (TMS)-activation based functional magnetic resonance imaging (fMRI) paradigms as well as functional connectivity in the resting-state^66–68^. Questions on mathematical modeling used to obtain ReHo (especially its non-linear origins) have been raised and clarified^30,32,50,69^. The adjusted power after control of covariates for the primary effects was robust (CRP-Glu prediction of MRS VOI ReHo = 0.81 and Subnetwork4 ReHo prediction of anhedonia = 0.94). Nevertheless, the sample size was relatively small, and inferences drawn from the study could also be limited by its cross-sectional design. We have provided effect size estimates with uncertainty (95% CI), applied bias-correction, corrected for multiple comparisons and wherever possible. Plasma CRP rather than other cytokines or CSF inflammatory markers was chosen for this study due to its overall stability, sensitivity and practical utility. Furthermore, as noted in another report from our group; plasma CRP demonstrated robust correlations with several other plasma inflammatory markers as well as CSF CRP justifying the focus on peripheral CRP ^70^.

In summary, the findings point to disruption of local and long-distance functional brain activity in a subgroup of patients with both high inflammation and basal ganglia glutamate. The study illustrates the possibility of combining blood and neuroimaging-based biomarkers relevant to the pathophysiology of depression to identify biologically homogenous subtypes for research and personalized treatments. If replicated, ReHo might also emerge as a measure of target-engagement in the brain to test efficacy of glial stabilizers (riluzole), glutamate modulators (memantine, lamotrigine) or anti-inflammatory agents (infliximab), especially in conditions where connectivity and network metrics are extensively compromised.

## Electronic supplementary material

Supplementary Information
