# Supplementary material for: Increased inflammation and brain glutamate define a subtype of depression with decreased regional homogeneity, impaired network integrity, and anhedonia
Source: Transl Psychiatry. 2018 Sep 10;8:189. doi: 10.1038/s41398-018-0241-4 (PMC6131242; doi:10.1038/s41398-018-0241-4)
Supplement: Supplementary file 1 — Supplementary Information [file 41398_2018_241_MOESM1_ESM.docx]

**Supplementary Information:**

| **Table of Contents** | **Page** |
| --- | --- |
| **Supplementary Table 1: Comparison of MRS data across the two groups.** | **2** |
| **Supplementary Table 2: Comparison of comorbid psychiatric disorders across the two groups** | **3** |
| **Supplemental Table 3: CRP-Glu group differences in local BOLD-oscillations.** | **4** |
| **Supplemental Table 3: ROIs based on ReHo difference maps.** | **5, 6** |
| **Supplementary Table 4:** **Variables associated with Subnetwork4 integrity.** | **7** |
| **Supplementary Figure 1: MRS Voxel** | **8** |
| **Supplementary Methods: Expanded methods section** | **9** |
| **Supplementary Results: Expanded results section** | **20** |

**Supplementary Table 1: Comparison of MRS data across the two groups**

| **Left Basal MRS voxel** | **Low CRP-Glu** | | | **High CRP-Glu** | | |  |  |  |
| --- | --- | --- | --- | --- | --- | --- | --- | --- | --- |
|  | **N** | **Mean** | **Std Dev** | **N** | **Mean** | **Std Dev** | **t(df)** | **p** |  |
| **Voxel Tissue Composition (mm^3^)** | | | | | | | | | |
| White matter  (% total voxel) | 20 | 3856.3 (44.8) | 399.61  (4.5) | 22 | 3911.00  (45.4) | 408.79  (4.7) | 0.43(40) | 0.66 |  |
| Gray matter  (% total voxel) | 20 | 4679.1 (54.4) | 385.53 (4.5) | 22 | 4646.86 (53.9) | 393.54  (4.6) | 0.27(40) | 0.79 |  |
| CSF Volume  (% total voxel) | 20 | 66.3  (0.7) | 53.23  (0.6) | 22 | 56.86 (0.7) | 43.46 (0.5) | 0.62(37) | 0.53 |  |
| Total Volume | 20 | 8601.7 | 62.92 | 22 | 8614.73 | 54.08 | 0.72(38) | 0.48 |  |
| **MRS Spectral Parameters** | | | | | | | | | |
| FWHM | 20 | 0.08 | 0.02 | 22 | 0.08 | 0.03 | 0.59(33) | 0.56 |  |
| SNR | 20 | 15.75 | 2.15 | 22 | 15.18 | 3.51 | 0.64(35) | 0.52 |  |
| Data Shift | 20 | 0.03 | 0.02 | 22 | 0.02 | 0.02 | 1.11(40) | 0.27 |  |
| CRLB (Glu) | 20 | 6.40 | 1.50 | 22 | 6.82 | 1.84 | 0.81(39) | 0.42 |  |
| **MRS Metabolites (absolute values corrected)** | | | | | | | | |  |
| Choline (abs) | 20 | 1.71 | 0.22 | 22 | 1.72 | 0.26 | 0.00(34) | 0.99 |  |
| Myo-inositol (abs) | 20 | 4.70 | 0.65 | 22 | 4.89 | 0.81 | 0.86(34) | 0.39 |  |
| NAA | 20 | 7.61 | 0.57 | 22 | 7.85 | 0.74 | 1.15(39) | 0.25 |  |
| Creatine | 20 | 7.85 | 0.69 | 22 | 7.66 | 0.72 | 0.00(40) | 0.99 |  |

**Abbreviations:** CSF: cerebrospinal fluid, FWHM: full-width half-maximum, SNR: signal-to-noise, CRLB: Cramer-Rao Lower Bounds, Glu: glutamate, NAA=n-acetyl aspartate

| **SCID IV Diagnosis** | **Low CRP-Glu** | | | **High CRP-Glu** | | |  |  |
| --- | --- | --- | --- | --- | --- | --- | --- | --- |
|  | **Total** | **# diagnosed** | **%** | **N** | **# diagnosed** | **%** | **ChiSq, (df)** | **p** |
| Depressed Bipolar I | 20 | 1 | 5.0 | 22 | 1 | 4.5 | 0.01 (1) | 0.94 |
| Depressed Bipolar II | 20 | 0 | 0 | 22 | 1 | 4.5 | 0.93 (1) | 0.33 |
| Depressed MDD | 20 | 19 | 95.0 | 22 | 20 | 90.9 | 0.26 (1) | 0.61 |
| Anxiety (combined) | 20 | 14 | 77.8 | 22 | 15 | 68.2 | 0.46 (1) | 0.50 |
| H/o substance use | 20 | 8 | 40.0 | 22 | 7 | 31.8 | 0.30 (1) | 0.58 |
| PTSD | 20 | 9 | 45.0 | 22 | 9 | 40.9 | 0.07 (1) | 0.58 |

**Supplementary Table 2:** **Comparison of comorbid psychiatric disorders across the Low vs High CRP-Glu groups.**

**Supplemental Table 3: CRP-Glu group differences in local BOLD-oscillations**

| **Left Basal Ganglia** | **Low CRP-Glu (n=20)** | | **High CRP-Glu (n=22)** | | **t(df)** | **p** |
| --- | --- | --- | --- | --- | --- | --- |
|  | Mean | Std Dev | Mean | Std Dev |  |  |
| ALFF | 503.57 | 71.89 | 485.34 | 62.98 | 0.87(38) | 0.39 |
| fALFF | 0.56 | 0.01 | 0.57 | 0.01 | 1.22(36) | 0.23 |
| mALFF | 0.77 | 0.05 | 0.79 | 0.05 | 0.92(39) | 0.36 |
| RSFA | 5.17 | 0.75 | 4.70 | 0.65 | 0.89(37) | 0.38 |
| fRSFA | 0.76 | 0.02 | 0.75 | 0.01 | 0.81(33) | 0.42 |
| lREHO | 0.15 | 0.03 | 0.13 | 0.02 | 2.6(34) | 0.01 |
| ReHo | 0.13 | 0.03 | 0.11 | 0.02 | 2.78(33) | 0.01 |

**Abbreviations:**

ALFF: amplitude of local fluctuations; fALFF: Fractional amplitude of local fluctuations; mALFF: Mean amplitude of local fluctuations; RSFA: Resting state fluctuation amplitude; fRSFA: Fractional Resting state fluctuation amplitude, lREHO: Low-Frequency Filtered ReHo. Only ALFF, RSFA and ReHo were used in the Canonical Discriminant Models to minimize collinearity and variable inflation.

**Supplemental Table 4: ROIs based on ReHo difference maps.**

ROIs were selected based on coordinates provided by previous studies on depression. 2) Ratio of overlapped volume between the 10-mm diameter sphere centered at the MNI coordinates and the ReHo difference map over the volume of a 10-mm diameter sphere. 3) ROIs in the same subgroup are coded in the same color as in Figure 3.

| **ROIs (Colored by networks)** | **MNI Coordinates** | | | **Overlap Ratio ^2^** |
| --- | --- | --- | --- | --- |
|  | x | y | z |  |
| **Subnetwork1** | | | | |
| **58, left Inferior Frontal, l-IF** | **-51** | **8** | **-2** | **0.7456** |
| **63, right Superior Temporal, r-ST** | **58** | **-16** | **7** | **0.7844** |
| **65, left Superior Temporal 1, l-ST1** | **-60** | **-25** | **14** | **0.8427** |
| **66, left Superior Temporal 2, l-ST2** | **-49** | **-26** | **5** | **0.7281** |
| **67, right Insula 1, r-Ins1** | **43** | **-23** | **20** | **0.699** |
| **69, left Inferior Parietal, l-IP** | **-53** | **-22** | **23** | **0.9203** |
| **73, left Insula 1, l-Ins1** | **-30** | **-27** | **12** | **0.6291** |
| **95, right Post Cingulate 1, r-PCC1** | **11** | **-54** | **17** | **0.7592** |
| **1004, left Insula 2, l-Ins2** | **-40** | **-14** | **16** | **0.9902** |
| **1005, right Cuneus, r-Cun** | **5** | **-83** | **13** | **0.7475** |
| **1008, right Fusiform, r-Fus** | **38** | **-52** | **-16** | **0.6834** |
| **1010, right Insula 3, r-Ins3** | **41** | **-15** | **5** | **0.9728** |
| **1016, right Temporal-Parietal junction, r-TPJ** | **51** | **-40** | **12** | **0.9456** |
| **Subnetwork2** | | | | |
| **94, left Posterior Cingulate 1, l-PCC1** | **-2** | **-37** | **44** | **0.6524** |
| **117, left Middle Temporal 1, l-MT1** | **-56** | **-13** | **-10** | **0.5844** |
| **118, left Middle Temporal 2, l-MT2** | **-58** | **-30** | **-4** | **0.5902** |
| **125, right Parahippocampal, r-PH** | **27** | **-37** | **-13** | **0.7844** |
| **133, left Posterior Cingulate 2, l-PCC1** | **-2** | **-35** | **31** | **1** |
| **212, left Anterior Cingulate 3, l-ACC3** | **-11** | **26** | **25** | **0.866** |
| **213, left Medial Prefrontal, l-MPF** | **-1** | **15** | **44** | **0.6058** |
| **215, right Anterior Cingulate, r-ACC** | **0** | **30** | **27** | **0.5223** |
| **221, right Posterior Cingulate 2, r-PCC2** | **2** | **-24** | **30** | **0.8116** |
| **239, right Middle Temporal, r-MT** | **51** | **-29** | **-4** | **0.9533** |
| **1009, right Insula 2, r-Ins2** | **33** | **22** | **-8** | **0.831** |
| **Subnetwork3** | | | | |
| **98, left Dorsal Superior Frontal, l-DSF** | **-10** | **39** | **52** | **0.6349** |
| **111, left Anterior Cingulate 1, l-ACC1** | **-11** | **45** | **8** | **0.9786** |
| **113, left Anterior Cingulate 2, l-ACC2** | **-3** | **42** | **16** | **0.8271** |
| **153, right Lateral Occipital, r-LO** | **43** | **-78** | **-12** | **0.7009** |
| **1003, right Supramarginal, r-SM** | **-60** | **-51** | **30** | **0.7669** |
| **1015, left VTA / SN, l-VTA/SN** | **-8** | **-10** | **-11** | **0.73** |
| **1017, left Anterior Prefrontal, l-APF** | **-4** | **58** | **14** | **0.998** |
| **Subnetwork4** | | | | |
| **109, left Ventral Medial Prefrontal, l-vMPFC** | **-3** | **44** | **-9** | **0.6194** |
| **233, right Basal Ganglia 1, r-BG1** | **15** | **5** | **7** | **0.73** |
| **1001, left Supplementary Motor Area, l-SMA** | **-1** | **6** | **67** | **0.9475** |
| **1002, left Dorsolateral Prefrontal, l-DLPFC** | **-48** | **20** | **38** | **0.9902** |
| **1006, right Lingual, r-Lin** | **11** | **-81** | **-18** | **0.9456** |
| **1007, right Cerebellum, r-Cere** | **36** | **-59** | **-29** | **0.9864** |
| **1011, right Basal Ganglia 2, r-BG2** | **21** | **2** | **17** | **0.9203** |
| **1012, right Basal Ganglia 3, r-BG3** | **20** | **8** | **-10** | **0.6796** |
| **1013, right Basal Ganglia 4, r-BG4** | **9** | **14** | **-2** | **0.5087** |
| **1014, left Basal Ganglia, l-BG** | **-14** | **11** | **-6** | **0.7961** |

**Supplementary Table 4: Variables associated with Subnetwork4 integrity.**

| **Independent** | **Dependent** | **F Ratio** | **FDRp** | **Partial**  **Eta Sq** | **d(95%CI)** |  |
| --- | --- | --- | --- | --- | --- | --- |
| **CRP-Glu contrast** | | | | | | |
| High-Low CRP-Glu | Radius | 5.76 | 0.03 | 0.13 | 0.91(0.13-1.67) |  |
| **ReHo x Network Associations** | | | | | | |
| Subnetwork4 ReHo | Diameter | 8.36 | 0.019 | 0.18 | 0.84(0.18-1.46) |  |
| Subnetwork4 ReHo | Path Length | 9.2821 | 0.013 | 0.20 | 0.90(0.22-1.53) |  |
| Subnetwork4 ReHo | Global Efficiency | 12.16 | 0.004 | 0.24 | 1(0.28-1.63) |  |
| Subnetwork4 ReHo | Strength Positivity | 7.56 | 0.027 | 0.17 | 0.90(0.20-1.5) |  |
| **Network x Behavior Associations** | | | | | | |
| Assortativity | 5CMT | 11.95 | 0.004 | 0.24 | 0.77(0.09-1.40) |  |

**Abbreviations:** FDR- false discovery rate, ReHo = regional homogeneity, 5CMT – 5-Choice Task (CANTAB) Movement Time.

**Supplementary Figure 1:** **MRS Voxel**

**Supplementary Methods:**

**Subjects:** Participants were recruited through local media outlets and mental health providers. Patients were 21–65 years old with a diagnosis of major depressive episode resulting from a unipolar or bipolar mood disorder using Structured Clinical Interview for DSM-IV (SCID).^1,2^ Medical conditions confirmed by medical history, laboratory testing, electrocardiogram and physical exam including uncontrolled cardiovascular, endocrinologic, hematologic, hepatic, renal, or neurologic disease, autoimmune conditions (i.e. rheumatoid arthritis, inflammatory bowel disease, multiple sclerosis, lupus), chronic infection (i.e. HIV, hepatitis B or C), history of liver abnormalities or cancer, or evidence of infection within one month of screening that required antibiotic or antiviral therapy were considered exclusionary. Pregnant or lactating females were excluded from the study. Other exclusionary criteria included a history of schizophrenia (determined by SCID-IV); active psychotic symptoms of any type; substance abuse and/or dependence within the past 6 months (determined by SCID-IV); an active eating disorder or obsessive-compulsive disorder; active suicidal ideation determined by a score of 3 or higher on item #3 of the 17-item Hamilton Depression Rating Scale (HAM-D); and/or a score of less than 28 on the Mini-Mental State Examination.^3^ Of note, subjects with mood-associated hyperphagic behavior were not excluded.

All patients were free of psychotropic medications for at least 4 weeks (8 weeks for fluoxetine) and had not taken any medications known to affect the immune system [e.g. glucocorticoids, statins, angiotensin II inhibitors, and nonsteroidal anti-inflammatory agents (NSAIDs) excluding aspirin 81 mg/d] within the past 6 months (2 weeks for NSAIDS). Other medications were allowed as dictated by their treating physicians. All participants signed informed consent, and the study was approved *a priori* by the Institutional Review Board of Emory University and registered in ClinicalTrials.gov (NCT01426997). Blood was collected at two screening visits spaced 1 to 4 weeks apart for analysis of CRP at the Emory University Hospital Clinical Laboratory. Any measurement >10 mg/L was repeated at ~2-week intervals to ensure stable levels of CRP over time, and in combination with laboratory tests and physical examination, to exclude participants with active infections or other acute or unstable medical conditions. Study procedures occurred in the same order over 2 days with MRI scans on Day 1 and blood sampling and behavioral assessments on Day 2. Data from this sample have been included in previous reports.^4^

**Behavioral Assessments:** The different scales used for behavioral ratings have been described extensively in our previous publication.^4^

1. Depression severity was measured using Inventory of Depressive Symptoms–Self Reported (IDS-SR).^5,6^ The IDS-SR is comprised of 30 questions encompassing a range of symptom severity scored 0-3. Anhedonia was measured using a 3-item subscale derived from IDS-SR items #8, 19 and 21 (IDS3) that has been previously validated in other studies.^4,7,8^

**Cognitive Assessments**

1. Motor speed was assessed using Finger Tapping Test.^9^;
2. Psychomotor processing speed was measured using Trails Making Test A (TMT)^10^;
3. Digit Symbol Substitution Test (DSST) of Wechsler Adult Intelligence Scale^11^;
4. Movement and Reaction times were measured using Reaction Time Task of the Cambridge Neuropsychological Test Automated Battery (CANTAB).^12,13^ The task included Simple Reaction Time task that yielded performance scores in terms of motor speed (simple movement time or SMT) and reaction time (simple reaction time or SRT respectively) and Five-Choice Reaction Time (5CMT, 5CRT respectively). It also included a more complex version i.e., a Five-Choice Reaction Time task that provided performance scores in terms of motor speed (five-choice movement time or 5MT) and reaction time (five-choice reaction time or 5RT respectively).
5. Cognitive processing speed was measured using “Stockings of Cambridge” (SOC) Tasks of CANTAB that involved manipulating colored balls into a predefined space (socks) on the display screen. Measures included number of problems solved on first choice, mean choices to correct, mean latency (speed of response). Of note, the SOC provided test performance scores along 3 dimensions - ‘mean initial thinking time’, ‘mean moves’ and ‘mean subsequent thinking time’ - calculated for 2, 3, 4, 5 moves each. To reduce complexity of multiple measures principal components (SOC1, SOC2, SOC3) were extracted to reflected each of the 3 dimensions and were used for further analyses.

**CRP:** Blood was obtained in the morning (between 8-10AM) in EDTA tubes through an indwelling catheter after participants had at least 30 minutes of rest. Blood was immediately centrifuged (1000g for 15 minutes at 4°C), and plasma was removed and stored at −80°C until batched assay as described earlier.^4,8^ Hs-CRP was measured by the immunoturbidometric method, using the Beckman AU 480 chemistry analyzer and the Ultra WR CRP reagent kit (Sekisui Diagnostics, LLC, Lexington, MA, USA). Mean inter- and intra-assay coefficients of variation were reliably <10%.

**MRI:** Neuroimaging data was acquired on a 3T Magnetom Trio scanner (Siemens Medical Solutions USA) with a 12-channel head coil at the Emory-GA Tech BME Biomedical Imaging Technology Center in the afternoon (3PM 2 hours).

**Anatomical T1 images** were obtained using three-dimensional magnetization- prepared rapid gradient-echo with 176 1-mm-thick sagittal slices with the following parameters: repetition time (TR)=2300 milliseconds (ms), echo time (TE)=3.02 ms, time following inversion (TI)=1100 ms, flip angle=8^o^ and voxel size 1×1×1 mm^3^.

**Single voxel MRS data** were acquired with TR=3000 ms, TE=30 ms, sampling size=1024, 128 averages. One voxel sized 17×30×17 mm^3^ located on the left basal ganglia was used to obtain single- voxel ^1^H-MRS as previously reported.^4,14^ Spatial localization was implemented using PRESS technique.^15^ Numerically optimized Shinnar-Le Roux (SLR) radio frequency pulses were used for PRESS (90°,180°,180°).^15^ Four unsuppressed water FIDs (free induction decay) was acquired for eddy current suppression and phase correction ^16^. The FWHM of unsuppressed water peak was optimized to <25 Hz by shimming. The axial view was used to verify voxel placement and that the area covered included most of caudate, putamen, pallidal, thalamic and white matter regions but excluded insula in the lateral (axial view) and ventricle in superior/medial planes (axial and sagittal views - Supplemental Figure 1).

**Resting-state fMRI images** were acquired using a Z-saga EPI-pulse sequence for recovering ventral-frontal signal losses regularly seen in gradient-echo BOLD fMRI.^17^ Z-saga images were acquired at 3.4x3.4x4 mm resolution in 30x4-mm-thick axial slices with the following parameters: FOV=220mm, TR=2950 ms, TE1/TE2^=^30/67 ms, FA=90^o^, scan time=7.4 min (150 repetitions). Subjects were required to look at a fixation cross during the scan. Subjects were not allowed to eat or consume caffeine 3 hours prior or to smoke cigarettes 1 hour prior to the scan and all subjects reported compliance.

**Analysis of MRS data - estimation of absolute glutamate concentrations**: MRS metabolites were estimated with water-scaling using LC Model using following settings: spectral bandwidth 0.2-4.0 ppm and 2048 complex points.^18^ The entire 19-metabolite basis set provided by LC Model prior-knowledge fit algorithm was entered. MRS data with high metabolite variance [Cramer-Rao Lower Bounds (CRLB)>20] were excluded. Estimation of the absolute metabolite concentrations in a voxel of interest (VOI) is affected by the parcellation ratios of GM, WM and CSF (water) in the VOI. Absolute quantitation of glutamate was corrected for partial volume effects using previously validated methods.^19-22^ Briefly, T1 images were used to generate GM, WM and CSF segments using FreeSurfer (<https://surfer.nmr.mgh.harvard.edu>).^23^ VOIs were re-constructed within individual subject’s T1 structural images based on the size and location for the left BG VOI. The GM, WM and ventricle / CSF masks were applied to the VOIs to evaluate the volumes of these three segments in the VOIs and ratios of GM, WM and CSF were then calculated. All these preprocesses were conducted with AFNI (https://afni.nimh.nih.gov).^24^ The absolute glutamate concentrations generated by the LCModel was corrected for CSF using the formula:

C=C_o_ × 1/(1−f_CSF_),

where C is the corrected metabolite concentrations, C_o_ is the metabolite concentrations generated by LCModel output and f_CSF_ is the fraction of the CSF volume.^4^ The analysis was focused on left basal ganglia glutamate as the primary hypothesis focused on this region.

**Hierarchical cluster-based CRP-Glu grouping:** Agglomerative Hierarchical Cluster Analysis (HCA) using Ward' s linkage method in JMP Pro v13 for Mac (SAS Institute, Cary, NC, USA) was used to classify subjects based on plasma CRP and MRS-based measures of basal ganglia Glu. Both values were log-transformed to scale for uniformity and normality. Cubic Clustering Criterion (CCC— a measure of the cluster numbers providing the best fi t) and evaluation of screen plots were used to inform the number of groups. Both CCC (-1.891 for 2 cluster solutions vs. -2.88, -2.61, -2.13 for 3, 4, and 5 cluster solutions, respectively) and screen plot revealed a two-cluster solution as the most parsimonious model. A dendrogram depicting the cluster differentiation is provided in Fig. 1 b. '' Variable clustering" in JMP Pro (similar to '' varclus'' in SAS) was also used to identify groupings of variables based on shared brain signatures.

**Preprocessing of resting state time-series data:** For each subject, preprocess procedure was performed with AFNI following these steps: 1) estimation of outlier fraction for each TR (the image volume at the TR with minimum outliers in the entire time series was used as the base in motion correction and T1-EPI registration), 2) removal of signal spikes, 3) slice-timing shift correction, 4) motion correction, 5) registration between T1 and EPI images, 6) transformation of the lateral ventricle mask (eroded by one voxel) into the EPI space, and 7) removal of noise signals with a generalized linear models (GLM) analysis in AFNI. Using GLM procedure, the following signals were regressed out: 12 motion parameters (demeaned and derivatives of 6 motion time series generated from motion correction), and the first 3 principal components of the lateral ventricle (CSF) signals. After regressing out the motion and CSF-related signals, regional activity data including ReHo were calculated for the residual time series derived from the GLM. WM signals were included in the analysis as stated in the body of the text. Of note, ReHo metrics included both GM and WM signals, because WM is a key target of inflammation and glutamate dysregulation.^25-28^

**Local BOLD oscillatory measures in basal ganglia MRS volume-of-interest (VOI):** Local BOLD oscillatory measures including ReHo, amplitude of low frequency fluctuations (ALFF) and resting state fluctuation amplitude (RSFA) were calculated in the left basal ganglia MRS VOI. ReHo is a voxel-wise metric that estimates the concordance in spontaneous BOLD activity between a given voxel and spatially adjacent neighbors within the context of a given time series.^29,30^ ALFF is an index measure that estimates amplitude of BOLD fluctuations in low frequency range (<0.01 - 0.1hz). ^31,32^ RSFA measures combine square root of power spectrum to determine vascular reactivity information contained in the regional intensity of spontaneous BOLD fluctuations of resting fMRI signal.^33^ For each subject, averaged values of all 3 BOLD-metric families (ReHo/ALFF/RSFA) and their fractionated derivatives were calculated and compared across all voxels within each left basal ganglia VOI (Supplemental Information –Table S1).

**Analysis of ReHo Data:** The ReHo data were analyzed in two ways: 1) VOI-based analysis: For each subject, the ReHo data were averaged across all voxels in the ACC and left BG VOIs, respectively. These VOI-ReHo values were then used for further group analysis. 2) ROI-based analysis: For each subject, the brain-only T1 images were aligned to the standard MNI space and using both linear and nonlinear registration methods. The ReHo data was then registered with the T1 images and converted into the MNI space with spatial smoothing (FWHM = 4 mm). Low-High CRP-Glu group contrast testing using t-test (AFNI “3dttest++”) was applied to the ReHo data now registered to the MNI space. This data was used to generate a ReHo difference map following application of corrections for multiple testing-induced false positive errors using “Clustsim” option of “3dttest++” options in AFNI (voxel-level p <= 0.01, cluster-wise p <= 0.05).^34,35^

**Location of ROIs:** ReHo is a functional imaging-based signal and approximating voxel-based functional BOLD imaging inputs to anatomically-constrained brain spaces has been met with some controversy ^36^. To overcome some of these problems, several recent attempts have provided exhaustive functional imaging based parcellation schemes. Regions of interest (ROIs) within the ReHo difference map were identified and selected using 264 functional network node (ROIs) prescriptions obtained from recently published exhaustive, functional parcellation scheme ^36^. Additional ROIs demonstrating significant associations with depression but missed by the aforementioned 264-node functional network parcellation system were curated from other meta analytic studies focused on depression and added within the ReHo difference map.^37,38^ MNI coordinates provided by these publications were used to place 10-mm diameter spheres. Using the logical operator “AND” in AFNI, 10-mm spheres with >50% overlap between sphere volume and ReHo difference map were identified and included as ROIs for further analysis. Only overlapping volumes between ReHo contrast and 10-mm spheres were considered. Of note, ROIs included only regions within the overlapping volumes between ReHo contrast and 10-mm spheres. Then ROI-masks were applied to calculate ReHo of a single or groups of ROIs (‘mean ReHo’) for each subject and averaged across the two study groups - High vs Low CRP-Glu status.

**Connectivity analysis among ROIs with ReHo differences:** Network integrity was investigated using connectivity matrices that measured connections between ROIs demonstrating ReHo alterations using two methods. Firstly, pairwise connectivity strength between ROIs were computed to calculate connectivity ‘z’ scores followed by network analysis based on graph theory.^39,40^ For each subject, the whole-brain residual time series data were temporally filtered (0.01 – 0.1 Hz), converted into the MNI space and spatially filtered (FWHM = 4 mm). Then, average time series were calculated for the 41 ROIs, respectively. All these steps were performed with AFNI. After that, cross-correlation analysis among the 41 time series data was preformed using MATLAB (MathWorks, Natick, MA, USA), generating a correlation coefficient matrix for each subject. The correlation coefficient matrix was further transformed into the Fisher z-score matrix. Graph-theory-based measures of global and local brain connectivity were obtained using Brain Connectivity Toolbox (<https://sites.google.com/site/bctnet>) on the MATLAB platform.^39,40^ Networks are a collection of nodes (vertices), and links (edges) between pairs of nodes, and for purposes of this study ReHo ROIs were modeled as “nodes”. Weighted, undirected measures of network efficacy were obtained using adjacency and distance matrices. The following network definitions obtained from the brain connectivity toolbox were used:

1. Degree and Similarity: ‘Node strength’ is the sum of weights of links connected to the node.
2. Clustering and Community Structure: ‘Clustering coefficient (CC)’ is the degree to which a node’s neighbors are also neighbors of each other (i.e., the “cliquishness” of a network neighborhood); ‘Transitivity’ is a normalized variant of CC and ‘Modularity’ is the existence of multiple distinct high-modularity partitions of the same network;
3. Assortativity and Core Structure: ‘Assortativity’ measures tendency for nodes with similar degrees to cluster with each other - indicating decreasing nodal resilience and diversity;
4. Paths and distances: ‘Path length’ measures shortest topological lengths of paths between all pairs of nodes and ‘Global Efficiency” is the inverse of shortest path length in the network, reflecting efficiency; and
5. Eccentricity (shortest topological path length between a node and any other given node in the network): ‘Diameter’ is maximum, and ‘Radius’ is the minimum eccentricity.

These graph theory metrics enabled computation of network-level as opposed to individual node-level measures. In addition, ‘average connectivity z-score’ were also calculated across groups of ROIs analysis by averaging the time series for further analysis.

**Statistical Methods:** Data were checked for univariate and multivariate normality using standard procedures. Comparisons of background variables were performed using t tests and chi square, and variables that were different between High and Low CRP-Glu groups were controlled as covariates. Linear, stepwise, discriminant function analysis (DFA) was used to predict membership in High vs Low CRP-Glu groups using a categorical classifier (x variable=CRP-Glu status) on known continuous variables (‘y’ responses). DFA has been used to effectively profile peripheral immune and neuroimaging changes associated with behavioral phenotypes and disorders.^41,42^ A Generalized Regression (GR) – adaptive elastic regression technique – was used to test predictive associations between ReHo measures in the identified ROIs and behavioral constructs (JMP Pro for Macintosh Version-13, SAS Institute, Cary, NC). By shrinking model coefficients to decrease prediction variance and by limiting effects of collinearity, the GR algorithm decreases the prediction error. The variables selected by the elastic net models were then imported into standard least squares regression models to enable estimation of parameter strength; to test for multiple comparisons (using False Discovery Rate corrected-p values or FDRp) and to measure statistical power and estimate effect sizes (eta squared and Cohen's d). Path analysis was performed using SEM protocol in STATA (College STATA (College Park, Texas, USA). All data were checked for univariate and multivariate normality using standard procedures (Shapiro-Wilk’s testing, Mahlanobis distance testing, etc.). Comparisons of background variables were performed using t tests and chi square, and variables that were different between groups were controlled as covariates.

**Linear, stepwise, discriminant function analysis (DFA)** was used to predict membership in High vs Low CRP/Glu groups using a categorical classifier (x) variable (CRP/Glu status) on known continuous predictors (‘y’ responses). DFA has been used to effectively profile peripheral immune and neuroimaging changes associated with behavioral phenotypes and disorders.^41,42^ The stepwise selection option was used for variable selection that best discriminated between groups. Following verification of univariate/multivariate normality and lack of prohibitive linear dependencies; stepwise DFA (Wilks’ entry criteria probability of F=0.05, removal criteria probability of F=0.10) was used to construct linear discriminatory model using all BOLD oscillatory measures (ALFF, RSFA, ReHo) that were associated with High/Low CRP/Glu group status. The accuracy of the variable selection algorithm (validation) was assessed using both hold-one-out cross-validation (to estimate model accuracy in future data sets that did not contribute to the construction of the prediction model) and Akaike’s Information Criteria (AIC) (to test and verify better fit estimates and model parsimony). Results provided in the main text are drawn from the post hoc ANOVA. To minimize the number of comparisons, independent Canonical Stepwise Linear Discriminant Function Analysis (DFA) followed by post hoc univariate ANOVA for each of the three domains of interest - mood and behavior, cognition and individual IDS-SR items - were used to identify variables associated with CRP-Glu groupings. The item ‘mood and behavioral’ items included mood scores of all scales including IDS-SR. A separate analysis for individual IDS-SR items was also used to identify relevant behavioral changes missed by pooling scores and to avoid collinearity after excluding IDS-SR subclassifiers for ‘diurnal variation’ #9A-B and the either/or classifier for appetite #11-12 due to scoring asymmetries.

**Generalized Regression (GR) - Elastic Net Regression** with AICc validation was used to test predictive associations between ReHo measures in the identified ROIs and behavioral constructs using JMP Pro for Macintosh Version 13 (SAS Institute, Cary, NC, USA). By shrinking model coefficients to decrease prediction variance and by limiting effects of collinearity (of note, all variable inflation factors<2) the GR algorithm decreases the prediction error. The variables selected using the GR method were then imported into standard least squares regression models to enable estimation of parameter strength before and after control of covariates (such as sex, BMI); to test for multiple comparisons (using False Discovery Rate corrected p values or FDRp), and to measure statistical power and estimate effect sizes (Cohen's d).

**Power calculations** were computed using G*Power.^43^

**Path analyses** were conducted using Structural Equation Modeling (SEM) model in STATA. The pathway from Subnetwork4 ReHo → Subnetwork4 measure → IDS3 was examined, with ReHo as exogenous and network measures as endogenous factors. To avoid distortion resulting from missing values, distribution-free aysmptotic models were used for combined (both), and High and Low CRP-Glu groups. Discrepancy between the fitted model and null were tested using ChiSq tests for saturation. The discrepancy between saturated and model was significant then a modification index was run to examine the sources of discrepancy. Direct, indirect and total effects were tested from this model following examination of the structural equations.

**Supplementary (Detailed) Results:**

**Sample Characteristics:** Of the 50 subjects recruited, eight were excluded (5 with poor quality MRS data due to excess motion artifacts or CRLB values >20%; 2 for whom behavioral or immune data were not available and one subject for whom resting state fMRI was not available]. Data from the remaining 42 patients were included in the analyses. **Figure 1** illustrates the analytic pipeline used for this study. Using HCA, two clusters - High (n=22) versus Low (n=20) CRP-Glu groups were identified and contrasted (**Figure 1B, Table 1**). Significant (High-Low) CRP-Glu group differences for both plasma CRP (mg/L) and absolute left basal ganglia absolute glutamate concentrations (mmol/kg) [3.60(2.58-4.60), t(df)=7.39(32), p<0.001 and 0.78(0.23±1.32), t(df)=2.86(40), p=0.007 respectively] validating the clustering algorithms (Figure 1). The samples were well matched on clinical and demographic variables excluding BMI, which was significantly higher BMI among High vs Low CRP-Glu (Table 1). Hence, BMI was controlled in all subsequent analyses (unless otherwise specified).

**Behavioral & Cognitive Variables:** IDS3 (adjusted R-Sq=0.10, F(1,40)=5,34, p=0.03), 5CMT (adjusted R-Sq=0.13, F(1,40)=7.29, p=0.01), TMT (adjusted R-Sq=0.09, F(1,40)=5.20,. P=0.03) and SOC1 (adjusted R-Sq=0.13, F(1,40)=7.19, p=0.01) were significantly associated with CRP-Glu Status **(Table 1)**. Analysis of individual IDS-SR items indicated that Item#8 (“Response of your mood to good or desired events”: adjusted R-Sq=0.13, F(1,40)=5.53, p=0.02) and item#30 (Leaden Paralysis/Physical Energy: adjusted R-Sq=0.14, F(1,37)=5.80, p=0.02) were also associated CRP-Glu status. The magnitude of differences was mostly in the ‘large’ range (Cohen’s d(95%CI) Low-High CRP-Glu; IDS3 [0.71(1.33-0.08)], 5CMT [0.83(1.46-0.20], TMT [0.70(1.33-0.07) and SOC1 [0.83(1.47-0.18)], Item#8 of IDSSR “Mood reactivity to positive events” [0.61(-0.02-1.22) and Item#30 Leaden Paralysis/Physical Energy [0.79(0.16-1.42)].

**Effect of CRP-Glu status on BOLD signal metrics in MRS voxel:** DFA with post hoc ANOVA identified reductions in ReHo in the left basal ganglia MRS VOI as the sole, local BOLD oscillatory measure associated with High CRP-Glu status (Wilks’ Lambda= 0.80, F(1,40)=10.20, p=0.003, canonical r=0.45; with leave-one-out cross-validation indicating a classification-accuracy of 76.2% (**Figure 2, Supplementary Table 2**). Owing to a lack of similar association, ALFF and RSFA were excluded from further analysis. Further analysis was conducted to clarify if the association between CRP-Glu status and ReHo within the MRS VOI was attributable to either CRP or glutamate alone or the combined effects of CRP with glutamate. Adaptive elastic net generalized regression models including all three prediction probabilities with covariates (age, sex, race, BMI, smoking) confirmed that neither CRP nor glutamate (both p>0.05) but only CRP-Glu status [Low-High contrast = PE=0.83(0.34-1.33), Wald ChiSq=10.8, p=0.001] predicted ReHo decreases. Of note, the same model also revealed that race [African Caucasian-American=-0.57(-1.08- -0.06), Wald ChiSq=4.82, p=0.03] had a significant impact on ReHo decreases and hence was controlled as a covariate in all subsequent analyses.

**Whole-brain ReHo and ReHo subnetwork partitioning:** To examine if CRP-Glu status was associated brain-wide ReHo changes, whole-brain ReHo-difference maps were used to contrast between the High and Low CRP-Glu groups (**Figure 3, Supplementary Table 3**). ReHo changes were noted in several regions and in both directions but only regions with decreased ReHo survived corrections for multiple comparisons. To reliably locate ReHo changes within functionally defined space, 10-mm spheres (=283) were placed on published functional coordinates and the volumes within spheres demonstrating > 50% overlap with ReHo-difference map were included as ROIs. A total of 41 ROIs - 27 from the first set 264 ROI ^36^ and an additional set of 17 ROIs curated from depression-related studies^37,38^ that fulfilled this criteria were included for further analysis. Variable clustering approaches were used to decompose the large number of ROIs (#41) into subgroups of ROIs that reflected subnetworks of identical ReHo profiles. Such operator-independent, data-driven group selection approaches have been used by other groups to cluster subjects according to shared signatures of brain dysfunction in depression ^38^. Using JMP Pro v13, that uses a process similar to “varclus” procedure in the SAS, (SAS Institute, Cary, NC) the 41 ROIs were decomposed into 4 subnetworks of 13, 11, 7 and 10 ROIs - Subnetwork (SNW) 1, 2, 3 and 4. A detailed description of the ROIs including their coordinates and subnetwork affiliations are provided in (**Supplementary Table 3**) ROI masks were applied within each of the four subgroups to calculate ‘mean ReHo” of each subnetwork for each individual and used for further analyses. Mean ReHo values of all 4 subnetworks were entered into penalized regression models to test associations with behavior and graph theory measures of network integrity.

**Subgroup4 ReHo and behavioral associations:** To minimize Type1 errors, omnibus analysis testing linear associations between mean ReHo of all 4 ReHo subnetworks along with 2 covariates (BMI, race) and 6 behavioral variables (IDS3, 5CMT, TMT, SOC1 and IDS-SR Items#8 and 30) were tested within a single model correcting for multiple corrections using FDR. Subnetwork4 mean ReHo and race survived multiple corrections (FDRp=0.01 each) within this model and were further into a recursive linear model analysis after excluding other variables to avoid variable inflation and collinearity. Subgroup4 ReHo was negatively associated with severity of anhedonia as measured as measured by both IDS3 scores both IDS3 and [PE=-20.8(-32.1- -9.63), t=-3.76, FDRp<0.001, d=1.22(0.55-1.84)] and IDS-SR Item#8 of [PE=-18.1(-30.1- -6.09), t=-3.05, FDRp=0.004, d=1.03(0.33-1.63]. Of note, Subnetwork4 included several ROIs within canonical reward circuitry including ventromedial prefrontal (vMPFC), and dorsal (DS) and ventral striatal (VS) regions^37,38,44^

**Functional correlates of ReHo changes:** Connectivity matrices between all 41 ROIs were used to examine if ReHo changes were associated with compromised network connectivity and integrity using multiple approaches. A visual inspection of the heatmap of connectivity z-scores between ROIs indicated significant reductions in clustering among High compared with Low CRP-Glu group (**Figure 4 A, B**). Striking group High vs. Low group differences were further noted on comparing group-averaged, z-transformed connectivity matrices using both correlation- and covariance-based matrix comparison approaches (Jennrich ChiSq(820)=2235.65 and Box ChiSq(861)=2645.61 respectively, both p<0.001). Graph theory-based measures global network integrity were obtained by modeling 41 ROIs as nodes in a putative network. All of these 11 network measures were entered into a single MANOVA for group comparisons after controlling for BMI and race. Following Benjamini-Hochberg correction for multiple comparisons post hoc analysis indicated reduced ‘global efficiency in High vs Low CRP-Glu groups (mean(95%CI)=0.22(0.20-0.24) vs. 0.25(0.23-0.0.27), t(34)=-2.10, p<0.04) further confirmed network impairment in the High CRP-Glu groups.

**Disruptions in network sub-network (Subgroup4) architecture associated with ReHo Changes:** Subgroup4 mean ReHo was associated with positively with network strength [positive connection strength, PE±SE=0.45±0.17, t=2.75, FDRp=0.009)] and network centricity [global efficiency=056±0.16, t=3.49, FDRp=0.001; path length=-0.51±0.17, t=-3.05] and with negatively with network eccentricity [diameter (-0.51±0.18, t=-2.89, p=0.006) and radius (0.40±0.17, t=2.36, p=0.02) only in Low but not High CRP-Glu groups. Assortativity (tendency for similar degrees to cluster with each other) was positively associated with increased movement time on 5CMT [FDRp=0.004, 0.77(0.09-1.40)]. Network eccentricity measures (diameter and radius) also positively predicted severity of scores on IDS-SR item#30 (paralysis/physical energy, FDRp<0.001 for both measures). Subnetwork4 assortativity was also associated with psychomotor slowing indexed by prolongation of 5CMT [PE=0.47, t=3.46, FDRp=0.004, d=0.77(0.09-1.40)]. Network eccentricity measures i.e., diameter and radius (reflecting greater nodal segregation) positively predicted severity of scores on IDS-SR item#30 ‘paralysis’ [PE=1.01, t=3.55, FDRp<0.001, d=1.18(0.60-1.84) and PE -1.02, t=-3.6, FDRp<0.001, d=1.24(0.55-1.88) for diameter and radius respectively]. Details are provided in **Supplementary Table 4**.

**Exploratory path analysis linking CRP-Glu, ReHo, Connectivity and Behavior:** An exploratory analysis was undertaken to examine if CRP-Glu status disrupted the relationship between local (ReHo) and broader network integrity and if this disrupted relationship was linked to the severity of anhedonia. Of the various network measures only network measures of eccentricity (p<0.005, Cohen’s d of 0.97-1.03), robustly differentiated between groups. Hence, a path analysis based on Structural Equation Modeling was used examine the links between all above findings. The pathway from Subnetwork4 ReHo → Subnetwork4 measure → IDS3 was examined independently in the combined and high and low CRP-Glu groups (**Figure 4C, D**). Network eccentricity demonstrated meaningful mediation effects when the groups were combined (indirect effect p=0.64 and 0.30 for diameter and radius respectively). However,

significant mediation effect of the path was seen only in the Low CRP-Glu group for both diameter [path coef(95%CI)=0.14(0.33-0.24), z=2.58, p=0.01] and radius [0.13(0.006-0.26, z=2.06, p=0.40)] but not among High CRP-Glu groups [p=0.99 and p=0.98 for diameter and radius respectively].

**Supplemental References:**

1. APA. *Diagnostic and statistical manual of mental disorders* IV edn. American Psychiatric Press, Inc: Washington, DC, USA. , 2000.

2. First MB, Spitzer RL, Gibbon M, Williams JB. *Structured Clinical Interview for DSM-IV*. American Psychiatric Press: Washington DC, 1997.

3. Folstein MF, Folstein SE, McHugh PR. "Mini-mental state". A practical method for grading the cognitive state of patients for the clinician. *J Psychiatr Res* 1975; **12**(3)**:** 189-198.

4. Haroon E, Fleischer CC, Felger JC, Chen X, Woolwine BJ, Patel T *et al.* Conceptual convergence: increased inflammation is associated with increased basal ganglia glutamate in patients with major depression. *Mol Psychiatry* 2016; **21**(10)**:** 1351-1357.

5. Rush AJ, Trivedi MH, Ibrahim HM, Carmody TJ, Arnow B, Klein DN *et al.* The 16-Item Quick Inventory of Depressive Symptomatology (QIDS), clinician rating (QIDS-C), and self-report (QIDS-SR): a psychometric evaluation in patients with chronic major depression. *Biol Psychiatry* 2003; **54**(5)**:** 573-583.

6. Trivedi MH, Rush AJ, Ibrahim HM, Carmody TJ, Biggs MM, Suppes T *et al.* The Inventory of Depressive Symptomatology, Clinician Rating (IDS-C) and Self-Report (IDS-SR), and the Quick Inventory of Depressive Symptomatology, Clinician Rating (QIDS-C) and Self-Report (QIDS-SR) in public sector patients with mood disorders: a psychometric evaluation. *Psychol Med* 2004; **34**(1)**:** 73-82.

7. Ameli R, Luckenbaugh DA, Gould NF, Holmes MK, Lally N, Ballard ED *et al.* SHAPS-C: the Snaith-Hamilton pleasure scale modified for clinician administration. *PeerJ* 2014; **2:** e429.

8. Felger JC, Li Z, Haroon E, Woolwine BJ, Jung MY, Hu X *et al.* Inflammation is associated with decreased functional connectivity within corticostriatal reward circuitry in depression. *Mol Psychiatry* 2016; **21**(10)**:** 1358-1365.

9. Collyer CE, Broadbent HA, Church RM. Preferred rates of repetitive tapping and categorical time production. *Percept Psychophys* 1994; **55**(4)**:** 443-453.

10. Reitan RM. The relation of the trail making test to organic brain damage. *J Consult Psychol* 1955; **19**(5)**:** 393-394.

11. Wechsler D. *Wechsler Adult Intelligence Test: Fourth Edition.* Pearson: San Antonio, TX, USA. , 2008.

12. Robbins TW, James M, Owen AM, Sahakian BJ, Lawrence AD, McInnes L *et al.* A study of performance on tests from the CANTAB battery sensitive to frontal lobe dysfunction in a large sample of normal volunteers: implications for theories of executive functioning and cognitive aging. *J Int Neuropsychol Soc* 1998; **4**(5)**:** 474-490.

13. Sahakian BJ, Owen AM. Computerized assessment in neuropsychiatry using CANTAB: discussion paper. *J R Soc Med* 1992; **85**(7)**:** 399-402.

14. Haroon E, Woolwine BJ, Chen X, Pace TW, Parekh S, Spivey JR *et al.* IFN-alpha-induced cortical and subcortical glutamate changes assessed by magnetic resonance spectroscopy. *Neuropsychopharmacology* 2014; **39**(7)**:** 1777-1785.

15. Bottomley PA. Spatial localization in NMR spectroscopy in vivo. *Ann N Y Acad Sci* 1987; **508:** 333-348.

16. Klose U. In vivo proton spectroscopy in presence of eddy currents. *Magnetic resonance in medicine : official journal of the Society of Magnetic Resonance in Medicine / Society of Magnetic Resonance in Medicine* 1990; **14**(1)**:** 26-30.

17. Heberlein KA, Hu X. Simultaneous acquisition of gradient-echo and asymmetric spin-echo for single-shot z-shim: Z-SAGA. *Magn Reson Med* 2004; **51**(1)**:** 212-216.

18. Provencher SK. LCModel & LCMgui User’s Manual. LCMODEL Inc: Oakville, ON, Canada, 2016, pp 1-174.

19. Jansen J, Backes W, Nicolay K, Kooi M. 1H MR Spectroscopy of the Brain: Absolute Quantification of Metabolites. *Radiology* 2007; **240**(2)**:** 318-332.

20. Kreis T, Ross B. Absolute Quantitation of Water and Metabolites in the Human Brain. 1. Compartments and Water. *J Magn Reson* 1992; **Series B**(102)**:** 1-8.

21. Shattuck DW, Sandor-Leahy SR, Schaper KA, Rottenberg DA, Leahy RM. Magnetic resonance image tissue classification using a partial volume model. *Neuroimage* 2001; **13**(5)**:** 856-876.

22. Gasparovic C, Yeo R, Mannell M, Ling J, Elgie R, Phillips J *et al.* Neurometabolite concentrations in gray and white matter in mild traumatic brain injury: an 1H-magnetic resonance spectroscopy study. *J Neurotrauma* 2009; **26**(10)**:** 1635-1643.

23. Fischl B. FreeSurfer. *Neuroimage* 2012; **62**(2)**:** 774-781.

24. Cox RW. AFNI: Software for analysis and visualization of functional magnetic resonance neuroimages. Software available at <https://afni.nimh.nih.gov>. *Computers and Biomedical Research* 1996; **29:** 162-173.

25. Alexopoulos G, Morimoto S. The inflammation hypothesis in geriatric depression. *Int J Geriatr Psychiatry* 2011; **26**(11)**:** 1109-1118.

26. Taylor WD, Aizenstein HJ, Alexopoulos GS. The vascular depression hypothesis: mechanisms linking vascular disease with depression. *Mol Psychiatry* 2013; **18**(9)**:** 963-974.

27. Benedetti F, Poletti S, Hoogenboezem TA, Mazza E, Ambree O, de Wit H *et al.* Inflammatory cytokines influence measures of white matter integrity in Bipolar Disorder. *J Affect Disord* 2016; **202:** 1-9.

28. Benedetti F, Yeh PH, Bellani M, Radaelli D, Nicoletti MA, Poletti S *et al.* Disruption of white matter integrity in bipolar depression as a possible structural marker of illness. *Biol Psychiatry* 2011; **69**(4)**:** 309-317.

29. Zang Y, Jiang T, Lu Y, He Y, Tian L. Regional homogeneity approach to fMRI data analysis. *Neuroimage* 2004; **22**(1)**:** 394-400.

30. Li Z, Prudente CN, Stilla R, Sathian K, Jinnah HA, Hu X. Alterations of resting-state fMRI measurements in individuals with cervical dystonia. *Hum Brain Mapp* 2017; **38**(8)**:** 4098-4108.

31. Zang YF, He Y, Zhu CZ, Cao QJ, Sui MQ, Liang M *et al.* Altered baseline brain activity in children with ADHD revealed by resting-state functional MRI. *Brain Dev* 2007; **29**(2)**:** 83-91.

32. Zou QH, Zhu CZ, Yang Y, Zuo XN, Long XY, Cao QJ *et al.* An improved approach to detection of amplitude of low-frequency fluctuation (ALFF) for resting-state fMRI: fractional ALFF. *J Neurosci Methods* 2008; **172**(1)**:** 137-141.

33. Kannurpatti SS, Biswal BB. Detection and scaling of task-induced fMRI-BOLD response using resting state fluctuations. *Neuroimage* 2008; **40**(4)**:** 1567-1574.

34. Eklund A, Nichols TE, Knutsson H. Cluster failure: Why fMRI inferences for spatial extent have inflated false-positive rates. *Proc Natl Acad Sci U S A* 2016; **113**(28)**:** 7900-7905.

35. Cox RW, Chen G, Glen DR, Reynolds RC, Taylor PA. FMRI Clustering in AFNI: False-Positive Rates Redux. *Brain Connect* 2017; **7**(3)**:** 152-171.

36. Power JD, Cohen AL, Nelson SM, Wig GS, Barnes KA, Church JA *et al.* Functional network organization of the human brain. *Neuron* 2011; **72**(4)**:** 665-678.

37. Kaiser RH, Andrews-Hanna JR, Wager TD, Pizzagalli DA. Large-Scale Network Dysfunction in Major Depressive Disorder: A Meta-analysis of Resting-State Functional Connectivity. *JAMA Psychiatry* 2015; **72**(6)**:** 603-611.

38. Drysdale AT, Grosenick L, Downar J, Dunlop K, Mansouri F, Meng Y *et al.* Resting-state connectivity biomarkers define neurophysiological subtypes of depression. *Nat Med* 2017; **23**(1)**:** 28-38.

39. Rubinov M, Sporns O. Complex network measures of brain connectivity: uses and interpretations. *Neuroimage* 2010; **52**(3)**:** 1059-1069.

40. Sporns O. *Networks of the Brain*. The MIT Press: Cambridge, MA, 2011.

41. Cagnin A, Brooks DJ, Kennedy AM, Gunn RN, Myers R, Turkheimer FE *et al.* In-vivo measurement of activated microglia in dementia. *The Lancet* 2001; **358**(9280)**:** 461-467.

42. Collado-Hidalgo A, Bower JE, Ganz PA, Cole SW, Irwin MR. Inflammatory biomarkers for persistent fatigue in breast cancer survivors. *Clin Cancer Res* 2006; **12**(9)**:** 2759-2766.

43. Faul F, Erdfelder E, Lang A, Buchner A. G*Power 3: a flexible statistical power analysis program for the social, behavioral, and biomedical sciences. *Behav Res Methods* 2007; **39**(2)**:** 175-191.

44. Haber SN, Knutson B. The reward circuit: linking primate anatomy and human imaging. *Neuropsychopharmacology* 2010; **35**(1)**:** 4-26.
